# THE INFLUENCE OF THE UNIVERSAL TWO-CHILD POLICY ON CHINA’S FUTURE AGING AND POPULATION

**DOI:** 10.1093/geroni/igz038.2596

**Published:** 2019-11-08

**Authors:** Can Jia, Handong li

**Affiliations:** 1 Systems Science, Beijing Normal University, Beijing, China, China; 2 Beijing Normal University, Beijing, China

## Abstract

China’s aging situation is becoming more and more prominent, and both the people and the government are facing unprecedented pressure of providing for the aged. For this reason, the Chinese government began implementing a new family planning policy for couples to have two children since 2016 (referred to as “universal two-child policy”). In order to explore the impact of the newly released policy, our research is based on the sixth census of China. And first, we use the cohort-component method and a Leslie matrix to construct the population prediction model. Considering some certain unique factors in China, such as the significant urban-rural dual structure and the household registration system and so on, we divide the total fertility rate into urban and rural areas which fully reflects the characteristics of China’s family planning policy. Then we predict and analyze the number and structure of China population between 2011 and 2050 based on the three scenarios of high, medium and low. And the results show that the Chinese population will present an inverted pyramid structure, and the population structure will continue to deteriorate. Besides, we adapt three indicators to analyze the aging trend in China, namely, the old-age coefficient, the population aging index, and the social dependency ratio. And the three indicators of China will continue to grow under the universal two-child policy with different changing rate, which means, the newly released policy will not change China’s aging population growth trend and the severity of China’s aging.

